# Reaching at-risk women for PrEP delivery: What can we learn from clinical trials in sub-Saharan Africa?

**DOI:** 10.1371/journal.pone.0218556

**Published:** 2019-06-21

**Authors:** Kayla Stankevitz, Katie Schwartz, Theresa Hoke, Yixuan Li, Michele Lanham, Imelda Mahaka, Saiqa Mullick

**Affiliations:** 1 FHI 360, Durham, NC, United States of America; 2 Pangaea Zimbabwe AIDS Trust, Harare, Zimbabwe; 3 Wits RHI, Johannesburg, South Africa; Sefako Makgatho Health Sciences University, SOUTH AFRICA

## Abstract

**Introduction:**

ARV-based pre-exposure prophylaxis (PrEP) has the potential to avert many new HIV infections, yet little is known about how to reach women at high risk for HIV infection and motivate them to initiate PrEP. Clinical trials have succeeded in recruiting at-risk participants, evidenced by control arm HIV incidence ≥3% (defined by the World Health Organization as “substantial risk”). We examined experiences from HIV prevention trials to document recruitment strategies and identify practical, potentially effective strategies for reaching women in real-world PrEP delivery.

**Methods:**

We conducted semi-structured qualitative phone interviews with 31 staff from five countries who had worked on one or more of seven ARV-based HIV prevention clinical trials. Questions explored recruitment strategies used to reach women at risk of HIV and to successfully communicate about PrEP (inclusive of oral and vaginal formulations). We structurally coded data in NVivo and analyzed codes to derive themes. We conducted results interpretation webinars with research and programmatic stakeholders to validate findings and develop recommendations.

**Results:**

Clinical trial researchers employed a range of recruitment strategies to recruit at-risk women. They recommended engaging the local community and potential PrEP users via community events, meetings with gatekeepers, and use of community advisory boards; and they encouraged interpersonal communication like presentations in waiting rooms and door-to-door recruitment to address personal concerns and prevent misinformation. Participants also stressed the importance of addressing the challenges that already exist within the health system to create a more enabling environment and delivering positive messages through a variety of communication channels to normalize PrEP.

**Conclusions:**

Findings from this study provide important insights into potentially effective ways for countries currently rolling out oral PrEP to reach at-risk women with information about PrEP and promote uptake.

## Introduction

Women in sub-Saharan Africa face a disproportionate burden of HIV: adult women comprise 56% of new adult infections, and over two thirds of new infections among young people are among young women [1–3]. Women have not fully benefited from prevention options like condoms and treatment as prevention due to underlying gender-power dynamics in many relationships [4–6], highlighting the need for continued efforts to develop appropriate and effective HIV prevention products for women and to deliver them at scale.

Recent trials show antiretroviral (ARV)-based HIV prevention products like topical microbicides, oral tablets, and vaginal rings (collectively referred to as pre-exposure prophylaxis, or PrEP) to be effective methods of HIV prevention among women [7–10]. As an HIV prevention method that women can use without a partner’s active involvement or consent, PrEP has the potential to prevent a considerable number of new HIV infections in women. The World Health Organization (WHO) recommends that oral PrEP should be a prevention choice for populations at substantial risk of HIV infection, defined as having a >3% incidence [11], yet little evidence exists on how to identify women at substantial risk and to stimulate their interest in oral PrEP use [12–14]. Localized incidence statistics are not readily available to identify populations who meet WHO’s definition of substantial risk. Further, programmatic rollout of oral PrEP is relatively new, and little evidence exists on strategies that have been successful at reaching women at substantial risk of HIV infection.

With ongoing questions on how best to identify young women at highest risk of HIV infection and limited real-world programmatic experience to examine, clinical trials testing PrEP provide a valuable source of lessons learned about PrEP delivery. Many have successfully identified and recruited women at substantial risk for HIV, as demonstrated by HIV incidence exceeding 3% in placebo arms [15], often substantially higher than population-wide incidence. For example, the MTN-003/VOICE trial (conducted in South Africa, Zimbabwe, and Uganda) had an overall HIV incidence of >5% per year in the placebo arm [16]. By comparison, the HIV incidence among South African women ages 15–49 at the time of the trial was 2.3% [17]. Of the women recruited and screened for the MTN-020/ASPIRE trial, 15% were not eligible due to seropositivity for HIV, indicating recruitment was reaching women at substantial risk for HIV [18].

Trial investigators have published on the process of implementing PrEP trials with women, reporting generally on recruitment methods [19, 20]. However, such publications typically lack practical “how-to” details. To tap into experience-based knowledge, this study explored strategies used in clinical trials for identifying high-risk women and recruiting them for trial participation. Acknowledging that clinical trial implementation differs from large-scale program delivery, the aim was to derive lessons from the clinical trial experience with potential relevance for application in real-world PrEP delivery.

## Methods

### Study design

We conducted a descriptive qualitative study to explore strategies used to reach at-risk women in clinical trials testing ARV-based prevention products (hereafter collectively referred to as “HIV prevention trials”). We completed a desk review to compile information about each HIV prevention trial. Then we conducted in-depth interviews with relevant project staff from completed and ongoing trials.

### Selection of HIV prevention trials

HIV prevention trials which met the following criteria were included: (1) phase IIb or III clinical trials that included ARV-based biomedical interventions for HIV prevention, (2) conducted in Africa, (3) enrolled HIV-negative female study participants, (4) started recruitment between January 2007- January 2017, and (5) had an overall HIV incidence rate of 3% or higher in the placebo arm.

### Participant recruitment

Research staff from eligible studies who were directly involved in designing, overseeing, or carrying out the recruitment activities were eligible to participate. To determine potential participants, we contacted study investigators for each trial and asked them to identify up to three relevant staff per study site. We attempted to contact all identified staff; those willing to participate and accessible via telephone or Skype were included in the sample.

### Data collection

Key informant interviews were conducted by phone or Skype between October 2017 and December 2017. A member of the research team facilitated each interview using a semi-structured interview guide while an assistant took detailed notes. Interviews were conducted individually or in small groups of up to three participants from the same research site. The interviewer explained the study and obtained verbal informed consent from all participants. All interviews were audio recorded. An interview guide was used to explore approaches used in the trials to reach at-risk women, perceived utility of approaches, lessons learned, and recommendations for incorporating approaches into real world PrEP delivery. If participants had experience working with multiple eligible HIV prevention trials, the interviewer encouraged the participant to discuss all trials, focusing on the most recent trial first.

### Data analysis

Detailed notes were reviewed and expanded following the interviews, and audio recordings were reviewed to elaborate notes and transcribe quotes [21]. Structural codes were developed based on the study objectives and interview questions, and emergent codes were developed based on new concepts emerging from the data [22]. Two people from the research team coded the detailed notes in NVivo 11 using a codebook. Another member of the research team reviewed the coding for consistency and re-coded as needed. The team then reviewed code reports and identified and summarized themes, along with illustrative quotes.

### Ethical considerations

This study was reviewed by FHI 360’s Office of International Research Ethics and deemed exempt from human subjects ethics review based on being low risk to participants.

## Results

### Trials and participants

The study team identified seven trials meeting study inclusion criteria (Table 1). These trials tested the following products in young women (age 18 to 45): 1% tenofovir vaginal (TFV) gel, oral tenofovir disoproxil fumarate (TDF), oral tenofovir–emtricitabine (TDF-FTC), and the dapivirine ring. Trials reported control arm HIV incidence rates from 3.1% - 9.1%.

**Table 1:** Trial characteristics.

Trial	Product	Phase	Trial Duration	Population (age)	Total Enrollment	Location	Control Arm Incidence*
CAPRISA 004 [23]	TFV gel	IIb	2007–2010	Women (18–40)	889	South Africa	9.1%
TDF2 [9]	TDF-FTC	III	2007–2011	Women and Men (18–39)	1219	Botswana	3.1%
MTN 003 (VOICE) [24]	TDF-FTC, TDF, TFV gel	IIb	2009–2012	Women (18–45)	5029	South Africa, Uganda, Zimbabwe	5.7%
FEM-PrEP [25]	TDF-FTC	III	2009–2013	Women (18–35)	2120	Kenya, South Africa, Tanzania**	5.0%
FACTS 001 [26]	TFV gel	III	2011–2014	Women (18–30)	2059	South Africa	4.0%
IPM 027 (The Ring study) [27]	dapivirine ring	III	2012–2016	Women (18–45)	1959	South Africa, Uganda	6.1%
MTN 020 (ASPIRE) [28]	dapivirine ring	III	2012–2015	Women (18–45)	2629	Malawi, South Africa, Uganda, Zimbabwe	4.5%

*HIV infection rate in control arm per 100 person-years

**Researchers in Kenya and Tanzania could not be reached for interview and did not participate in the study.

A total of 36 research staff from eligible trials were identified as potential participants. Four did not respond when contacted, and one declined. We conducted 17 interviews, one for each of 17 research sites, with 31 participants (Table 2). Most were women, and most worked on HIV prevention trials based in South Africa. Many participants had worked on more than one eligible HIV prevention trial: two participants had worked on three trials, and 13 participants were staff on two trials.

**Table 2:** Participant characteristics.

Characteristics	Number of informants
Sex	
Female	23
Male	8
Role	
Site PI	9
Study Coordinator	7
Community Manager	5
Recruitment Officer	10
Country	
South Africa	23
Uganda	3
Botswana	1
Malawi	2
Zimbabwe	2
HIV Prevention Trial*	
MTN 020 (ASPIRE)	14
MTN 003 (VOICE)	10
FACTS 001	10
IPM 027 (The Ring Study)	10
FEM-PrEP	2
CAPRISA 004	1
TDF2	1
TOTAL	31

*Some interviewees had experience with more than one trial.

Informants discussed strategies they used to reach women with PrEP, both in terms of how to reach women for recruitment purposes and how to effectively deliver and frame messaging around PrEP to motivate uptake.

### Strategies for reaching at-risk women with PrEP

Informants were asked which strategies they used to recruit at-risk women for trial participation. Our analysis revealed four primary approaches: community engagement, information dissemination, targeted recruitment, and clinician referrals. Specific strategies employed under each of these approaches are detailed in Table 3.

**Table 3:** Recruitment strategies employed in HIV prevention trials.

Strategy	Description	Number of sites that mentioned using strategy (n = 17)*
**Community engagement**		
Engagement meetings with gatekeepers	Before recruiting in the community, study teams visited gatekeepers-people who had influence within the community-to provide them information about the study and seek support.	15
Community advisory boards (CABs)	Study teams worked with advisory boards consisting of members of the community to provide information about the study, get information about the community, and ensure the research was community owned.	10
**Information dissemination**		
Community events	Recruitment staff attended local community events, such as health education events or World AIDS Day, and gave educational presentations, handed out study information sheets, and answered questions.	16
Posters and flyers	Recruitment staff hung posters and handed out flyers in public spaces and health facilities to educate the public about PrEP and promote the trial.	12
Media	Principal investigators and research staff were interviewed on local or national radio stations and studies ran television commercials to raise awareness about PrEP and promote the trial.	11
**Targeted recruitment**		
Presentations in waiting rooms	Recruitment staff were stationed in waiting rooms of clinics thought to serve at-risk populations to provide information and answer questions.	13
Word-of-mouth	Recruitment staff encouraged existing trial participants to promote participation among friends via word-of-mouth.	12
Venue-based recruitment	Recruitment staff provided information about the trial in areas where women congregate, such as: bars, brothels, shopping malls, communal taps, markets, or places on the street where people socialize.	10
Door-to-door	Recruitment staff provided door-to-door education about PrEP and the trial and discussed the details one-on-one with those who were interested.	8
**Clinician referral**		
Clinician referrals	Clinicians in family planning, STI, or HIV testing and counseling clinics were trained on PrEP and the trial by study staff and asked to educate and refer their clients.	14

* We did not probe specifically for each strategy, so additional sites may have used the strategy but not mentioned it.

#### Recruitment staff

Informants described the skills and experience they looked for when selecting recruitment staff. They felt prior experience working in the community was important, as this gave staff credibility and an understanding of the community. Recruitment staff were also expected to speak the same language as the community and to understand local culture and dialect, affording them the communication skills needed to actively engage with potential participants. Informants felt that strategies that gave potential participants a chance to interact one-on-one with a recruiter were the most effective. They stressed the importance of hiring people to whom potential participants could relate.

*"So if you want to recruit young women*, *you should have somebody who can speak their language*, *and know what their needs are*, *and understand their lifestyle*.*"*

When asked whether recruitment staff were all female, many study teams said it was important to have male staff as well, as some women would be interested in talking with men. Male recruitment staff also helped encourage male partner engagement in the studies and provide an extra level of security for female staff while they were out in the community.

Informants offered advice about who should be involved in outreach activities in real-world PrEP delivery settings and agreed that the traits important for recruitment staff were relevant outside the clinical trial context. Some suggested that community health workers or other trusted community-based workers could support community outreach and education.

#### Community engagement

All trials employed one or more community engagement approaches that involved educating individuals known as “gatekeepers” about PrEP and the trial. These were people within the targeted community regarded as having influence over both the study team’s access to prospective participants and women’s interest in joining the trial. Gatekeepers included community leaders such as chiefs, religious leaders, ward counselors, and non-governmental organization (NGO) representatives. Separately, studies often convened community advisory boards (CABs) consisting of community representatives who were charged with helping design and implement research being conducted in their community. Engagement with both gatekeepers and CABs sought to foster a collaborative atmosphere in which they could understand the trial and study product(s) and guide researchers in study promotion and recruitment. Often, they also provided approval for researchers to conduct community education and recruitment.

Informants found that engaging with community gatekeepers and CABs helped to create an enabling environment for the trial to take place and added legitimacy to the work. When gatekeepers spoke publicly in support of the research, it helped build trust in both the researchers and the study. While discussing the importance of community engagement, one informant said:

*"When you just walk up to somebody and tell them about a study [*…*] people often think you're coming with an agenda*. *If you get trusted community members who can speak about studies*, *its kind of takes some of the agenda away*.*"*

Since community gatekeepers and CAB members had access to different populations, engaging a wide range of people helped researchers to increase their reach.

*“I think that it’s difficult for us to reach everybody […] and each of these NGOs and different people reach different groups*. *So these help us to engage widely and get messages out*.*”*

When asked about real-world application, informants felt that engaging gatekeepers—including leaders, traditional healers, and senior people in the community—was essential. While CABs are not traditionally employed outside of the research context, they felt bringing together an advisory board around PrEP rollout in the community could be productive. Informants suggested stakeholders should have the chance to provide input to the rollout strategy to promote their ownership over the process. This information exchange should happen before PrEP promotion activities starts in the community.

#### Information dissemination

All study sites disseminated information to educate the community and potential study participants about clinical trial research generally, PrEP, and the study. Potential participants were not targeted with these approaches; rather, information was shared widely with the aim of creating a supportive environment for PrEP use and trial participation, thereby encouraging potential participants to self-identify and to seek out the study. Common channels for information dissemination included: speaking and handing out pamphlets at community events, distributing printed materials, and using mass media.

Our informants viewed information dissemination as essential as it allowed recruitment staff to reach many people simultaneously, raise awareness, and legitimize the study. Informants felt that hearing about the trial in the community before talking about it with a healthcare provider at the study clinic was helpful.

*"We learned that it is important to give information*, *especially way before you can introduce a product to the people*, *so that they know it exists*.*”*

Materials like posters and pamphlets posted in clinic waiting rooms and in the community were useful to allow participants time to educate themselves about PrEP, to learn about the study, and to consider participating.

*“By us giving them the pamphlets* …*it gave them some time to read through it*, *think about it*, *consider if they wanted to be part of such a program*.*"*

Media was a popular form of information dissemination, with radio being the most popular, followed by television. Participants said these were good ways of reaching large groups of people at once, as they would both raise awareness and legitimize the study. However, informants warned that wide-reaching information dissemination strategies lack personal interaction between potential participants and recruitment staff, and as such can lead to misconceptions or stigma. To avoid stigma, informants encouraged use of key messages rather than giving many details about target population or risk status.

*"On radio*, *we really don’t talk about “high risk*.*” We don't want to add stigma*. . . . *Instead of actively looking for a group*, *the group comes to us*, *and now we can prescreen one-on-one*.*"*

When considering translation to real-world PrEP delivery, informants felt information dissemination was equally important and strategies could easily be translated. They stressed that since young women are less likely to reach out and get information actively, rollout strategies to reach young women should employ multiple passive communication channels. Use of mass media in trials was limited, often due to the small scope of trials or ethical concerns, but informants expressed that outreach via media (including social media and nationally endorsed media channels) could build trust and combat stigma during PrEP rollout.

#### Targeted recruitment

Targeted recruitment of known or perceived at-risk groups-such as sex workers-within geographic areas of high prevalence was one of the most consistently employed and effective strategies mentioned by informants. Targeted recruitment allowed recruitment staff to have one-on-one or small group conversations with potential participants, allowing women who were contemplating joining the study to ask questions specific to their own situations, often privately. Informants agreed that these activities were crucial for reaching participants at-risk for HIV, as they increased interpersonal contact: by moving beyond the clinic, researchers could access people who do not normally seek health services.

Targeted recruitment was often guided by community mapping, which involved talking to gatekeepers, CABs, and other community members to understand areas where potential trial participants gather and which influential people to collaborate with. Informants stated that each community is different, and it is important to understand the community fully prior to recruitment.

*“First we did the community mapping and talked to community members to understand where the study could get a good number of women…*. *We visited the areas before the study*, *talked with the community members to know which areas to reach women*, *and got a sense of hotspots*.*”*

Some informants used existing data or conducted primary data collection as part of their community mapping. One team conducted in-depth interviews with gatekeepers and community members to understand the community and determine which places to target with recruitment efforts. Another team mapped recruitment areas of participants who became HIV-positive during the trial to identify areas with high risk; these areas were used for further recruitment.

*"In terms of statistics*, *we did map…*. *What we did is we said*, *‘Where are our sero-converters coming from*? *Which local districts*?*’ Then from there we gather these places are where we got our seroconverters…*. *And to date*, *those places still remain high risk*.*"*

Other study teams reported using existing information from other stakeholders. For example, one team partnered with NGOs who were conducting HIV testing in communities and asked them where most of their HIV-positive clients were coming from to determine hot spots. Another team worked with community members that were identified by community leaders to understand areas of high risk and provide door-to-door counseling in those neighborhoods.

Others found that existing data were not as helpful, because either they were not detailed enough, or prevalence was high enough that they felt the entire community was at risk. When discussing the use of statistics to find women most at-risk, one informant said:

*“The assumption is that everyone is at high risk*. *We have a prevalence of about 25%* …*so generally we think that if you are a woman and you live (here) you are high risk*.*”*

Using community mapping results, informants were able to target establishments where they were likely to find at-risk women, such as bars, brothels, youth clubs, and universities. Informants said this approach worked well when recruitment staff understood the community and were supported by community leaders, and could be employed in PrEP rollout if the same level of support was given. Other informants expressed concerns around privacy and safety, explaining that some potential trial participants were not comfortable talking to researchers while they were out with their friends. Further, sending recruiters out at night could be dangerous, and some teams had to hire secure vehicles for their recruiters to travel safely.

Another common approach was door-to-door recruitment. Research teams noted the importance of applying this method only in communities with high HIV prevalence or-in the absence of prevalence data-in communities perceived to be at-risk by community stakeholders. Going door-to-door in low prevalence areas was perceived to be neither time- nor cost-effective. Informants felt door-to-door was successful because it allowed recruiters to give each potential participant one-on-one attention and gave them time to answer questions.

*“With door-to-door you spend time with the person*. *You don't leave there until that person is clear about what you're talking about [*…*] unlike a group setting where you have limited time to explain and they may be afraid to ask personal questions*.*”*

Reflecting on translation to real-world delivery of PrEP, informants acknowledged that door-to-door recruitment is time-intensive. They stressed that every house must be visited to ensure PrEP use is not stigmatized by being associated with the visits made to the homes of people living with HIV. Some study teams reduced cost by partnering with other groups who were already providing door-to-door education or HIV testing services. Informants suggested this could be a strategy when applying this approach in non-trial contexts.

Informants also attempted to target known or perceived at-risk groups by encouraging recruitment among friends, spreading information about PrEP by word-of-mouth by enrolled trial participants. Several informants emphasized that this was particularly effective for recruiting high-risk participants, specifically participants who were similar to those already enrolled.

*“That works kind of both ways*. *If you find a high-risk participant*, *they are likely to have a high-risk friend*. *But also if you find a participant who is going to be poor with retention then their friend is also likely poor at retention*.*”*

Some informants reported experiencing challenges with trial participants giving their acquaintances the wrong information. To combat this, informants suggested giving out pamphlets or information sheets to share with people they tell about trial participation.

#### Clinician referrals

Most informants described recruiting participants from clinical settings, including family planning clinics, and sexually transmitted infection service settings. Study teams trained health care providers to support recruitment by giving them information about the trial, contraception (since using an effective contraceptive method was often required for participation), and PrEP. They reasoned that providers offering these services would have a good sense of a client’s trial eligibility.

*“The low-hanging fruits would be the place where people seek HIV testing-both facility and NGOs- HIV testing*, *STI*, *and cervical cancer*.*”*

While most respondents regarded this as an important approach for identifying prospective PrEP users in clinical trials as well as real world PrEP delivery, several informants highlighted challenges associated with relying on clinicians to promote PrEP trial participation among their clients. Some mentioned that this strategy may not work if at-risk women are not utilizing health services. Some study teams encountered challenges with provider workload, which led to fewer referrals than expected.

*“As the study went on*, *the clinics were too busy so that the referral became fewer and fewer*. *This was a challenge throughout*.*”*

Some study teams had problems with provider turnover and regularly went back to the clinics to review and train new clinical staff.

*“Health provider turnover was a challenge*, *because new health providers did not know about the study*. *So sometimes we went back to the facility for trainings*, *reviewing with health providers as well as giving training to new health providers*.*”*

### Messaging and communication strategies

In addition to discussing specific recruitment strategies, informants provided insight into how to best deliver and frame messaging around PrEP to motivate uptake in real-world PrEP delivery settings. This included using positive messaging, contextualizing messages, using several different channels of communications, and avoiding stigma.

#### Positive messaging

Discussing PrEP as a tool to empower women, rather than using fear-based messages about disease and risk, was described as an effective way of motivating study participation and PrEP uptake. Informants mentioned that many women were motivated to use PrEP to empower them to prevent HIV without permission from their partners. Informants commented that women often do not know their partners’ HIV status and lack the ability to insist on condom use. As a result, using messages about empowerment was successful in supporting recruitment. Informants also discussed messages that suggested altruistic motivations for trial participation:

*"Women knew that participating in a study that was […] for women*, *it gave power to negotiate safer sex if it worked*. *So the key statement would be*, *'We are looking for something for us*, *for women*. *We want to be the heroes of women*, *heroes for our children*, *heroes for our sisters*,*' like that*. *Because with condoms they know*, *'It is not always in my power*.*’”*

#### Messages should be grounded in the local context

Informants felt that people would be more likely to listen to messages about PrEP if the messages were grounded in the reality of the community. They stressed that PrEP promotional materials should be locally made and reflect local, easy to understand language. Often, sites were given posters or materials that were made at the national level, or even above national level. These materials did not resonate with the local audience. One informant described an experience of materials they were given with un-relatable stock photos that resulted in backlash at the community level.

Similarly, some informants felt national statistics were not compelling, but local statistics about HIV incidence and prevalence, that women can directly translate to the risk they face, were helpful.

*"I think it is important for people to get information and relate them to their situation or what is happening in the real world*. *Like being able to understand there is HIV*, *especially in their communities*. *And to understand that there is need for something to be done*. *By giving them this information*, *it will be important*.*"*

#### Use of multi-faceted communication channels

Informants stressed the importance of using multiple channels to deliver messages about PrEP, as hearing about PrEP via many sources can add legitimacy to the product. Informants encountered challenges during trials associated with insufficient publicity:

*"You find that when speaking to people* … *who have never ever heard of PrEP… they don’t believe that the PrEP is really available because they haven't heard about it on radio*, *they haven't seen it in the newspaper*, *it’s just us coming to them with pamphlets giving them information about PrEP*. *So I think that we should involve the media as much as we can*.*"*

As discussed above, informants used communication channels like radio, television, and public forums to disseminate information about PrEP and their trials. Informants suggested that using communication channels that focus not only on those who might participate in the study, but also on their partners and others in the community. Messages that inform partners and community leaders can help educate and create a supportive environment.

#### Avoiding stigmatization of PrEP

Many informants expressed concerns about framing messages about PrEP to focus on stigmatized populations, like sex workers or men who have sex with men. If messaging was designed to target specific populations, they warned, other populations might not feel comfortable taking it. Some researchers stated that they did target mainly sex workers but made sure not to frame their messages to indicate such.

Informants also expressed concerns with the term, “high risk.” There was a consensus that “risk” and “high risk” should never be used in PrEP messaging.

*"In the community*, *using ‘high risk’ words is stigmatizing*. *The challenge is finding the alternative to this word*. *Finding ‘high-risk women’ is finding [the] right language to speak with them without stigmatizing*.*”*

## Discussion

As countries rollout oral PrEP for HIV prevention, programs need strategies to identify those at substantial risk for HIV infection, reach them with messages about PrEP, and inspire them to consider initiating oral PrEP. Clinical trials included in this study used multi-pronged approaches to recruitment, though the exact combination of strategies differed. They began with community engagement and buy-in from stakeholders, which for most trials continued throughout the course of the study. Following initial community engagement, they used a combination of recruitment approaches, including general dissemination of information about the trial and product, targeted recruitment approaches within the community, and clinician referrals.

The clinical trial experiences documented in this study provide insights with implications for PrEP delivery outside the research context. Below we summarize recommendations from this work, developed in collaboration with PrEP clinical trialists and stakeholders involved in programmatic delivery of PrEP.

### Creating a supportive environment for PrEP use

The study results highlighted that HIV stigma could affect PrEP uptake and use. As countries move from clinical trials and demonstration projects to real-world oral PrEP delivery, it will be essential to engage communities to promote acceptance and minimize stigmatization of PrEP [29]. Given the impetus on trials to adhere to the Good Participatory Practice (GPP) Guidelines, it is not surprising that trials employed extensive community engagement strategies [30, 31]. A recent systematic review describes a range of techniques to engage communities in clinical trials [30]. Our results complement those findings by exploring previously unpublished experiences from trials specifically related to PrEP. This study highlights the importance of community engagement strategies, including engagement with gatekeepers and advisory boards, as one component of a demand creation strategy that can help create a supportive community environment for PrEP uptake and use outside of the clinical trial context.

Many informants reported concerns that PrEP was not widely understood, which could hinder uptake. The information dissemination approaches used in clinical trials, including speaking at community events, posting flyers, and use of media, can be employed as part of PrEP rollout strategies to help promote PrEP awareness. Further, informants suggested mass media should be used to reach potential users and the general population in and around communities where PrEP is available. This result is consistent with prior experience with female condom programming, in which it was discovered that widespread promotion to the community at large was needed to destigmatize the method and normalize it as a viable prevention option [32]. While no studies exist exploring the role of mass media in sensitizing the population on PrEP specifically, studies have found that using mass media has been an important strategy in promoting and normalizing other prevention services, including family planning [33, 34], HIV testing and counseling [35], and voluntary medical male circumcision [36]. This study suggests information dissemination approaches should be utilized to reach everyone, not just potential PrEP users, and help normalize this new prevention option.

### Reaching at-risk women with PrEP

This study demonstrated that clinical trials applied various community-based approaches to reach women at risk of HIV who could benefit from PrEP. Outreach was especially important for reaching marginalized populations who are less able to access health services. We found that targeted recruitment methods tailored to the needs of the community were very successful. For instance, informants in this study used techniques like door-to-door education and recruitment in shopping malls to reach young women who they felt were not accessing health services. PrEP implementers should examine targeted approaches employed by clinical trials, including mapping of high-risk recruitment areas, word-of-mouth, and piggybacking on existing door-to-door outreach, and assess whether these approaches would be appropriate to apply in their settings.

In addition to highlighting the importance of community-based strategies for reaching women with PrEP, our study affirmed the importance of promoting and providing PrEP in clinic-based settings. Many clinical trials successfully recruited from HIV testing centers, family planning clinics, and clinics providing STI services, suggesting that integrated service delivery in these settings should be considered for oral PrEP rollout. However, informants experienced many challenges in clinical trial recruitment that may hold true in programmatic rollout of PrEP, including challenges with provider workload, provider turnover, and quality of care. Research has revealed that lapses in delivery of added services like PrEP can be attributed to health system limitations and overwhelming demands placed on providers [37]. Interventions to integrate PrEP into existing services should pay close attention to health system barriers and, when needed, introduce remedial measures to improve the patient experience and ensure providers have the time and motivation to offer PrEP consistently.

### Messaging and communication about PrEP

These results add to a growing body of literature on how to best frame messages around PrEP [38, 39]. This study found that clinical trial researchers used positive messaging to promote PrEP uptake in trial settings. Informants felt that PrEP use was facilitated by promoting it as a tool to empower women to protect themselves and that fear-based messages about disease and risk should be avoided. Messages around PrEP should be designed to mitigate stigma, avoid labelling people as “high risk,” and avoid noticeably targeting stigmatized populations, such as sex workers. Further, developing messages grounded in the local context and using familiar vernacular and local statistics easily understood by the target population were all considered to be important approaches. Materials and messages should be pre-tested locally to ensure they resonate with the target audience.

Further, these results suggest that recruitment and outreach that uses direct interpersonal communication—via peer educators or outreach workers—could encourage greater uptake of this new HIV prevention option that is prone to misinformation and stigma. One-on-one communication allowed potential PrEP users to ask questions that they would not be comfortable asking in a group education setting. The use of peer educators has been shown to be a successful task-shifting strategy to provide effective education, counselling, and adherence support [40, 41]. Engaging peer educators and outreach workers in PrEP rollout could prove a successful strategy for delivering positive messages and promoting PrEP uptake.

## Limitations

The limitations of this investigation are acknowledged. Participants represent a purposive sample of research staff from selected trials that were suggested by the principal investigator and willing to participate. Due to this being a retrospective evaluation, recall bias may be present in participant responses. The study was purely descriptive and qualitative, and we have no means of evaluating the strategies by site or intervention other than the overall incidence rate of each trial. No data exist from trials to compare the effect of these various approaches on client recruitment. Further, we acknowledge that there are important differences between clinical trials and programmatic PrEP rollout that may affect the saliency of these findings in the real world. Clinical trials typically have relatively more financial and technical resources that allow more intensive participant engagement than is possible in standard public health programming. Ethical considerations posed challenges to using social media for recruitment in the trials included in this investigation; as such this study does not provide insight into social media strategies or experiences. The relevance of the findings to programs serving adolescent girls—currently a high-priority target population for PrEP—is limited since no eligible clinical trial enrolled participants under age 18. Despite these limitations, this investigation makes an important contribution by including trials testing a range of PrEP products across several countries, and findings were consistent across countries and in multiple settings. We held validation webinars to discuss the findings with both research staff and people involved in programmatic rollout of PrEP, and we used those insights to translate findings into recommendations for real world PrEP delivery. As such, we believe lessons learned from this work are important to inform future implementation of PrEP.

## Conclusion

Findings from this study address a critical gap in knowledge by providing insight into how to best reach at-risk women with information about PrEP and promote uptake. These recommendations can be used to inform implementation strategies that are community-specific and reach women in a variety of settings, including those who have not traditionally been reached with HIV prevention interventions.

## Supporting information

S1 File Interview guide. (DOCX)
