# Supplementary material for: Reaching at-risk women for PrEP delivery: What can we learn from clinical trials in sub-Saharan Africa?
Source: PLoS One. 2019 Jun 21;14(6):e0218556. doi: 10.1371/journal.pone.0218556 (PMC6588242; doi:10.1371/journal.pone.0218556)
Supplement: S1 File — (DOCX) [file pone.0218556.s001.docx]

**Reaching High Risk Women for PrEP: Learning from ARV-based HIV prevention trials**

**In-Depth Interview Guide**

**[Complete in-depth interview oral consent form]**

Thanks for taking time to speak with me today. This research aims to understand recruitment strategies for high risk women in HIV prevention trials. As governments start to rollout PrEP, it’s important that we understand how to reach this high-risk population. We’re hoping to learn what worked for you, what didn’t, and specifically how you feel the strategies you used could be modified for use in real-world service delivery of HIV prevention products.

| **Question** | **Probes** |
| --- | --- |
| I’d like to learn more about the recruitment strategies you used. We’ve found that most studies look both to the community and to clinics for recruitment. For community based recruitment, we mean things like community events, information sessions, word-of-mouth, radio shows, and texting- any kind of recruitment that took place outside of a facility. | |
| Can you summarize the overall approach used for community-based recruitment, or informing potential participants in the community about the trial? | 1. Did you engage with key population or community organizations (those working with FSW for example)? 2. What kinds of materials did you use to provide information to people? 3. Did trial participants play a role in supporting recruitment efforts? (referrals, word-of-mouth). 4. How did you determine the best locations for community-based recruitment events? |
| 1. We’re interested in how your experiences could be used while rolling out PrEP or other prevention products in real-world service delivery. How do you think the community based strategies we talked about could be used as part of PrEP rollout? | 1. What strategies would work best to reach young women? 2. How many people would you need to implement this as part of PrEP rollout? 3. What are some of the challenges you could face? 4. What community influencers or community groups were important to involve to enable recruitment of high risk women? |
| 1. Can you talk more about the role of community leaders, CAB, or other stakeholders in helping to recruit participants? | 1. What types of community leaders played a role? 2. What types of education or training did you provide them? 3. What experiences did you have working with community leaders that were not successful in helping to recruit trial participants? |
| 1. Can you summarize the overall approach you used for clinic based recruitment? | 1. What types of clinics did you recruit at? 2. What kinds of materials did you use to provide information to patients? |
| 1. Thinking about how health services are currently delivered in the public sector, what would we have to do to encourage high risk women to initiate PrEP in health facilities? | 1. How do we work with providers to make them effective advocates for PrEP? 2. What kind of education should be given to providers about PrEP? Why is this important?   . |
| 1. What messages were communicated to women to help them assess their risk of HIV and to consider taking PrEP? | 1. Tell me about any screening procedures that were used to assess behaviors and identify high risk women. 2. What screening procedures were used to identify women likely to be consistent PrEP users? 3. How could these procedures be used into real world service delivery? |
| 1. When thinking about a young women or adolescent girls specifically, would some of these recruitment methods work better than others? | 1. What things are important to keep in mind when engaging young women about HIV prevention? |
| 1. If you had one piece of advice about what the health sector could do to encourage high-risk women to seek PrEP services , what would it be? | 1. What are the key take-aways or lessons learned? 2. What advice do you have about reaching this population? 3. Is there anything else you’d like to tell me? |
